# Cooperation of imipramine blue and tyrosine kinase blockade demonstrates activity against chronic myeloid leukemia

**DOI:** 10.18632/oncotarget.10541

**Published:** 2016-07-12

**Authors:** Kamilla M.E. Laidlaw, Samuel Berhan, Suhu Liu, Giovannino Silvestri, Tessa L. Holyoake, David A. Frank, Bharat Aggarwal, Michael Y. Bonner, Danilo Perrotti, Heather G. Jørgensen, Jack L. Arbiser

**Affiliations:** ^1^ Paul O'Gorman Leukemia Research Centre, Institute of Cancer Sciences, College of Medical, Veterinary and Life Sciences, University of Glasgow, Gartnavel General Hospital, Glasgow, G12 0ZD, United Kingdom; ^2^ Department of Medical Oncology, Dana Farber Cancer Institute, Harvard Medical School, Boston, MA 02215, USA; ^3^ Department of Experimental Therapeutics, The University of Texas MD Anderson Cancer Center, Houston, TX 77030, USA; ^4^ Department of Dermatology, Emory University School of Medicine, Atlanta, GA 30322, USA; ^5^ Atlanta Veterans Administration Hospital, Atlanta, GA 30322, USA; ^6^ Department of Medicine, Marlene and Stewart Greenebaum Cancer Center, University of Maryland School of Medicine, Baltimore, MD 21201, USA

**Keywords:** chronic myeloid leukemia, tyrosine kinase inhibitor, imipramine blue, nilotinib, NADPH oxidase

## Abstract

The use of tyrosine kinase inhibitors (TKI), including nilotinib, has revolutionized the treatment of chronic myeloid leukemia (CML). However current unmet clinical needs include combating activation of additional survival signaling pathways in persistent leukemia stem cells after long-term TKI therapy. A ubiquitous signaling alteration in cancer, including CML, is activation of reactive oxygen species (ROS) signaling, which may potentiate stem cell activity and mediate resistance to both conventional chemotherapy and targeted inhibitors. We have developed a novel nicotinamide adenine dinucleotide phosphate (NADPH) oxidase inhibitor, imipramine blue (IB) that targets ROS generation. ROS levels are known to be elevated in CML with respect to normal hematopoietic stem/progenitor cells and not corrected by TKI. We demonstrate that IB has additive benefit with nilotinib in inhibiting proliferation, viability, and clonogenic function of TKI-insensitive quiescent CD34^+^ CML chronic phase (CP) cells while normal CD34^+^ cells retained their clonogenic capacity in response to this combination therapy *in vitro*. Mechanistically, the pro-apoptotic activity of IB likely resides in part through its dual ability to block NF-κB and re-activate the tumor suppressor protein phosphatase 2A (PP2A). Combining BCR-ABL1 kinase inhibition with NADPH oxidase blockade may be beneficial in eradication of CML and worthy of further investigation.

## INTRODUCTION

Genomic instability underpins cancer. Point mutations, double strand breaks and translocations arising from oxidative stress are commonplace with elevated reactive oxygen species (ROS) generated by mitochondrial respiration mediating such DNA damage [1, 2]. Genetic lesions are of particular significance in stem cells. Endosteal niches in which hematopoietic stem cells (HSC) reside provide a hypoxic protective environment conducive to maintaining low ROS and consequently high reconstitution function [3]. Quiescent HSC, despite their relatively high mitochondrial content, exist in a low energy state that restricts DNA damage from replication stress. Proliferating (self-renewing) HSC switch to a high energy state, whereby mitochondrial respiration generates ROS. Mitochondrial ROS can stimulate nicotinamide adenine dinucleotide phosphate (NADPH) oxidases [4]. Pharmacological targeting of this cycle of ROS production to neutralise its DNA damaging effects and limit genomic instability is an attractive proposition in cancer of stem cell origin, such as chronic myeloid leukemia (CML).

CML is a myeloid neoplasm characterized by the *BCR-ABL1* fusion oncogene, a constitutively active tyrosine kinase. Because this kinase is unique to tumor cells, it provided an attractive target for pharmacologic development of small molecule, orally bioavailable tyrosine kinase inhibitors (TKI) [5]. Imatinib was the first clinically used TKI, and the CML treatment paradigm led to the development of targeted therapies for other driver mutations, such as Braf in melanoma, and EGFR and Alk mutations in lung cancer [6]. While TKIs can control chronic phase (CP) CML for several years, progression through accelerated phase (AP) to terminal blast crisis (BC) can still occur [7]. Even maximal TK inhibition is incapable of completely eliminating CML stem cells [8]. Hamilton *et al*., noted that deprivation of growth factors and addition of the potent dual Src-Abl kinase inhibitor, dasatinib resulted in the enrichment of primitive stem cells capable of proliferation once dasatinib is withdrawn *in vitro* [9]. Although clinical resistance is a relatively rare event (in up to 20% of cases), many mechanisms including mutation of BCR-ABL1, especially of the ATP binding pocket [6] or activation of additional signaling pathways independent of BCR-ABL1have been identified in CML [5, 10].

The most primitive (lineage negative, CD34^+^ CD38^−/+^) leukemia stem and progenitor cells (LSC/LPC) from CP CML patients were found to have higher (2- to 4-fold) ROS levels compared to normal HSC, which were not corrected by inhibition of BCR-ABL1 kinase activity with TKI [11]. Interestingly, poor responders to TKI therapy were found to have higher levels of ROS in their LSC at diagnosis than good responders who went on to achieve complete cytogenetic response (CCR) and major/complete molecular response (MMR/CMR) [11]. Persistence of TKI-insensitive LSC under such long-term oxidative stress will risk expansion of dominant TKI-resistant clones and, perhaps, evolution of BC. Indeed G:C to A:T substitutions, as in the E255K and T315I TKI-resistant BCR-ABL1 kinase mutations, are commonly consequent of ROS-induced DNA damage [11]. Skorski's group identified electron leakage from mitochondrial respiratory chain complex III (MRC-cIII) as a major source of ROS mediated DNA damage in CML LSC and targeted its activity with an inhibitor of Rac2, a GTPase that can modify mitochondrial membrane potential and electron flow through MRC [12].

The ‘reactive oxygen driven' solid tumor has previously been described, characterized by high levels of superoxide generation in tumor cells [13] that are often refractory to conventional chemotherapy, targeted therapy and radiation [2, 14]. Previously we have demonstrated the efficacy of the synthetic NADPH oxidase inhibitor imipramine blue (IB) to block the invasion of glioblastoma multiforme (GBM) into the brain parenchyma [15], and therefore, prolong survival in animal models likely through eradication of ROS-driven GBM stem cells. As the role of ROS in hematopoietic tumors is clearly also now emerging [16, 17], we responded to the call for ROS inhibitors as novel therapies for CML. We hypothesized that IB could be suitable for use in CML, not just by limiting genomic instability and disease progression to BC, but moreover as a potential LSC toxic agent. In this paper, we demonstrated that IB curbs survival of CML LSC/LPCs, and that its effect was potentiated by co-treatment with TKIs (i.e. nilotinib). Mechanistically, we showed that the pro-apoptotic activity of IB likely resides in its propensity towards being a PP2A activating drug (PAD) [8]. Combination therapy with a NADPH oxidase inhibitor and nilotinib may help prevent emergence of TKI-resistance and/or neutralise TKI-insensitive CML LSC. This is achieved by blockade of LSC specific ROS signaling alongside quenching of oncoprotein activity, respectively. Such a dual approach may be applicable to other ROS-driven hematopoietic malignancies with different driver oncogenic fusions and associated genomic instability.

## RESULTS

### IB reduces numbers of viable primary CD34^+^CML^+^CP cells and BCR-ABL1^+^ human BC cell lines *in vitro*

**Figure 1:**
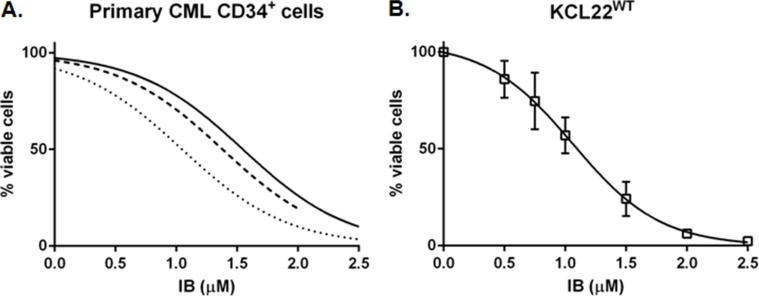
Imipramine blue (IB) decreases viable BCR-ABL1^+^ CML cell number in a concentration dependent manner with respect to untreated control (**A**) The number of viable CD34^+^ CML-CP cells remaining after 72 h in culture with increasing concentrations of IB, as counted by Trypan Blue dye exclusion method, was normalized to the No Drug Control (NDC) for each of three patients (solid line CML003; dashed line CML388; dotted line CML391). Minimum 5 data points per curve, curve fit *r*^2^ = 0.84, 0.97 and 0.94 for CML003, 388 and 391, respectively). (**B**) KCL22 cells with un-mutated (wildtype, WT) BCR-ABL1 were cultured with increasing concentration of IB and counted after 72 h (mean ± SEM of 3 repeat experiments).

We first wished to demonstrate clinically relevant activity of IB in CML cells, so primary CD34^+^ CML-CP cells and BCR-ABL1^+^ human CML-BC cell lines were incubated with the drug *in vitro* and total viable cell counts performed by Trypan Blue dye exclusion after 72 h. The IC50 of IB was similar in each of these cell contexts at 1.32 ± 0.25 μM for CD34^+^ CML-CP cells (*n* = 3) (Figure 1A), 1.13 ± 0.07 μM for KCL22WT cell line (Figure 1B), and approximately 1.65 μM for imatinib resistant KCL22T315I cells (Supplementary Figure S1A).

**Figure 2:**
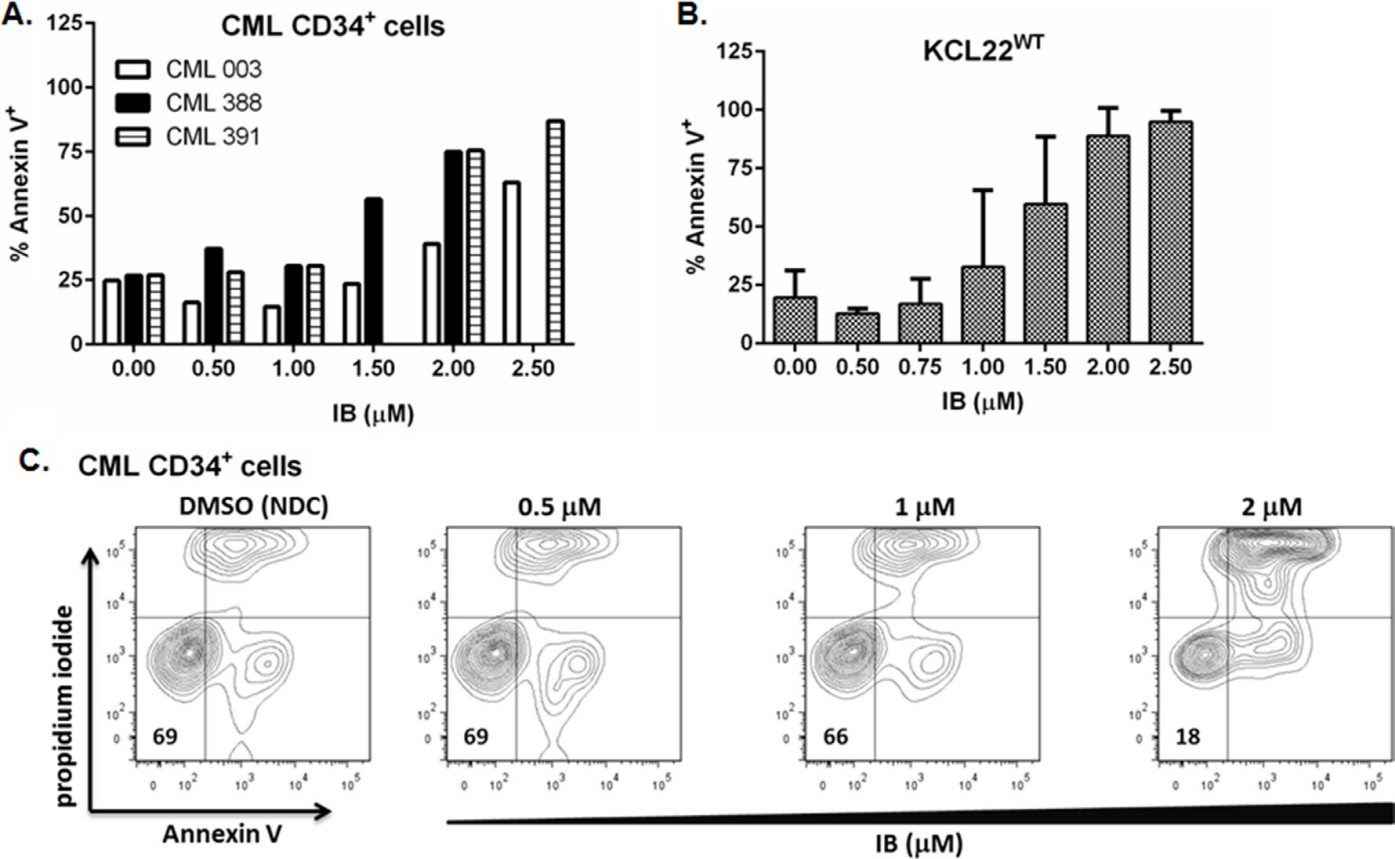
Apoptosis response of BCR-ABL1^+^ cells to imipramine blue (IB) IB treated (**A**) primary CD34^+^ CML-CP cells (*n* = 3), (**B**) KCL22WT cells (mean ± SEM; *n* = 3 experiments), were stained with Annexin V and propidium iodide to assess apoptotic response to the drug by flow cytometry. (**C**) Illustrative flow cytometry dot plots of primary CD34^+^ CML-CP cells (CML391) showing a concentration dependent apoptotic response to IB after 72 h.

Primary CD34^+^ CML-CP cells were more resistant to apoptosis induction by IB than BCR-ABL1^WT+^ CML-BC cell lines, nonetheless, the percentage of Annexin V^+^ cells increased in a concentration dependent manner in response to IB (Figure 2A–2C), and to a magnitude far greater than can be achieved with TKI. Collated data from flow cytometry experiments with 3 independent CD34^+^ CML-CP cell samples showed 26 ± 1 versus 63 ± 16 % Annexin V^+^ (NDC versus 2 μM IB; *p* = 0.04, Figure 2A). In our hands 3 μM nilotinib on average induces only about 5–10% apoptosis above background level in NDC (DMSO) in CD34^+^ CML-CP cells after 72 h *in vitro* (data not shown).

Interestingly apoptosis was not different from background level (NDC) in KCL22^WT^ cells at the respective IC_50_ for viable cell counts for IB (approx. 1 μM) which may suggest cell cycle arrest without kill at this concentration (Figure 2B). However just a small increase to 1.5 μM IB approximately doubled the level of apoptosis with respect to background (46 v 25 % Annexin V^+^, IB v NDC) in imatinib resistant KCL22T315I cells, whereas it was only 29% with 100 nM ponatinib, a next generation TKI to which these cells are sensitive (Supplementary Figure S1B).

### IB in combination with nilotinib inhibits proliferation of CD34^+^ CML-CP cells and is synergistic against BCR-ABL1^+^ cells *in vitro*

**Figure 3:**
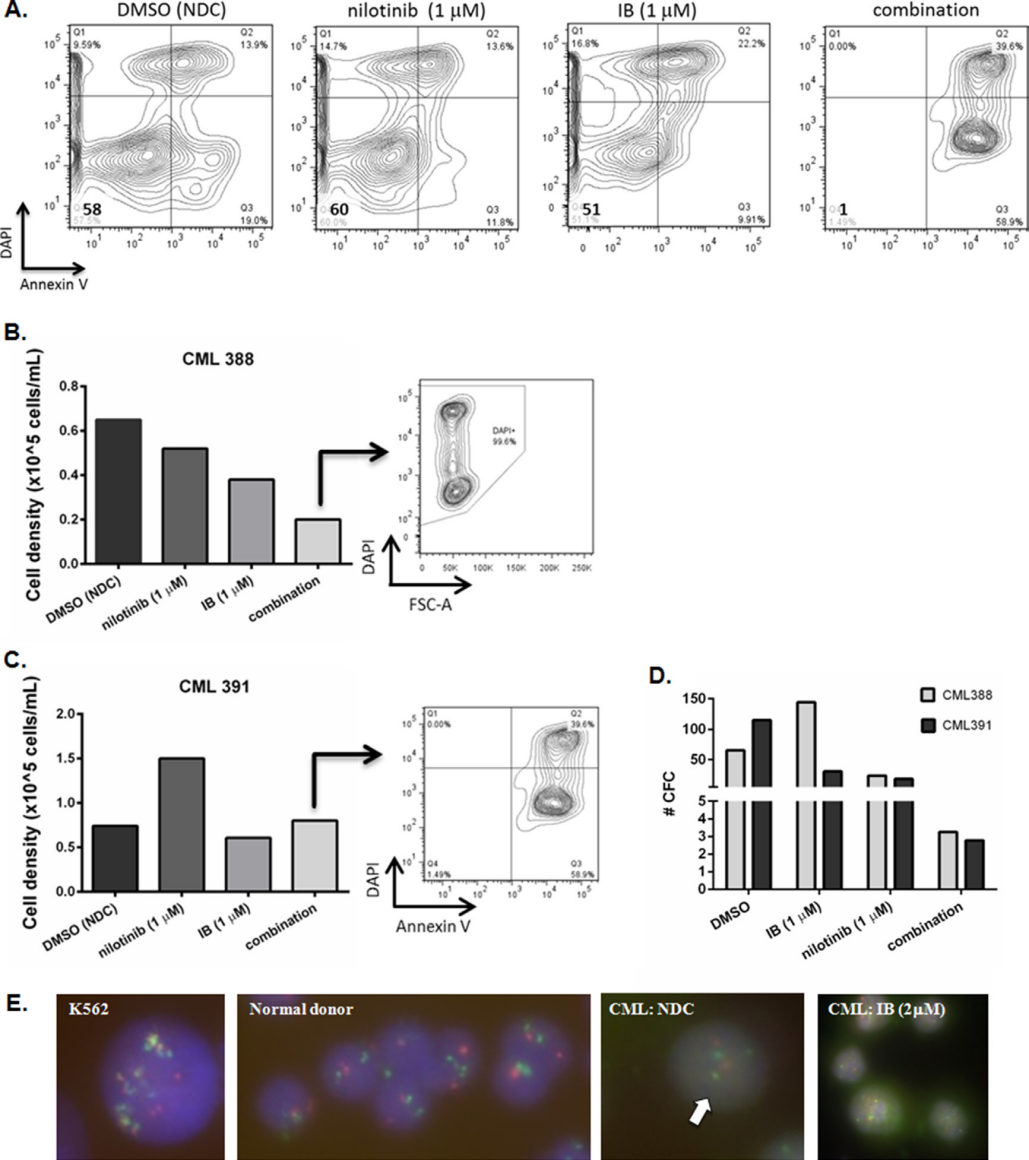
Response of primary CD34^+^CML-CP cells to imipramine blue (IB) in combination with nilotinib (**A**) Dot plots showing apoptotic response of CML391 to IB (1 μM), nilotinib (1 μM), or combination treatment for 72 h. (**B**, **C**) After 72 h treatment with drugs or diluent control DMSO /No Drug Control (NDC), primary CML-CP cells were harvested and counted (CML388 (B) and CML391 (C)). The cells were also stained with Annexin V and/or DAPI for viability. The dot plots shown are from wells treated with the drug combination illustrating that some cells counted under phase contrast microscopy and scored as viable owing to Trypan Blue exclusion are in fact committing to apoptosis and/or dying. (**D**) To assess their function after drug treatment, surviving cells were plated into semi-solid culture medium for 12 days after which time colony formation was scored for total number. (**E**) Fluorescence *in situ* hybridisation (FISH) for *BCR-ABL1* was performed on plucked colonies. Multiple yellow fusions were seen with K562 cells (positive control for FISH, ×100 magnification); 2 red and 2 green signals were evident in the normal donor (negative control for FISH, ×40 magnification); 2 red, 2 green, 1 yellow (fusion) signal as indicated by the arrow were seen in colonies plucked from CFC set up with CML-CP cells from an untreated well (CML388, ×100 magnification; this is an unusual but nonetheless positive fusion pattern). CFC from wells treated with 2 μM IB were leukemic i.e. BCR-ABL1 positive by FISH (×40 magnification).

We were interested to know if there was any combination effect between IB and TKI on BCR-ABL1^+^ cells *in vitro*. Primary CD34^+^ CML-CP cells were treated *in vitro* with nilotinib (1 μM) in combination with IB (1 μM). Induction of apoptosis with the combination of nilotinib plus IB was clearly markedly enhanced with respect to either drug as a single agent (representative example in Figure 3A) although this did not reach statistical significance owing to inter-patient variability in extent of response. It was noted that some cells counted under phase contrast microscopy and scored as viable owing to Trypan Blue dye exclusion were in fact committing to apoptosis and/or dying (Figure 3B and 3C). Hence it was important to assess their functional capacity (i.e. clonogenicity) after drug treatment. Surviving cells were plated into colony forming cell (CFC) assay. As each well started with the same cell number before drug addition, each had the same expansion potential, and so taking an equal fixed volume from each well to set up CFC at the end of a 72 h drug exposure experiment reflects drug activity in that time frame. It can be seen clearly that the few remaining CD34^+^ CML-CP cells could not divide to form colonies after combination treatment and so had been severely functionally compromised (Figure 3D). The colonies formed were BCR-ABL1^+^ by fluorescence *in situ* hybridization (FISH) (Figure 3E).

When assayed at fixed ratio according to the method of Chou and Talalay in BCR-ABL1^+^ K562^WT^ cells, nilotinib and IB in combination were synergistic with combination indices (CI) consistently less than 1 (Table 1).

**Table 1:** Determination of synergy for the imipramine blue (IB) plus nilotinib combination in K562 cells

nilotinib (μM)	IB (μM)	Fa	CI
0.0015	0.5	0.77	**0.958**
0.0015	1	0.96	**0.97**
0.003125	0.5	0.81	**1.008**
0.003125	1	0.98	**0.811**
0.00625	1	0.992	**0.642**
0.0125	0.5	0.94	**0.975**
0.0125	1	0.997	**0.507**
0.025	0.5	0.996	**0.417**
0.025	0.25	0.97	**0.806**

Fa Fraction affected; CI Combination Index.

### Cell division tracking of primary CFSE labelled CD34^+^ CML-CP cells

**Figure 4:**
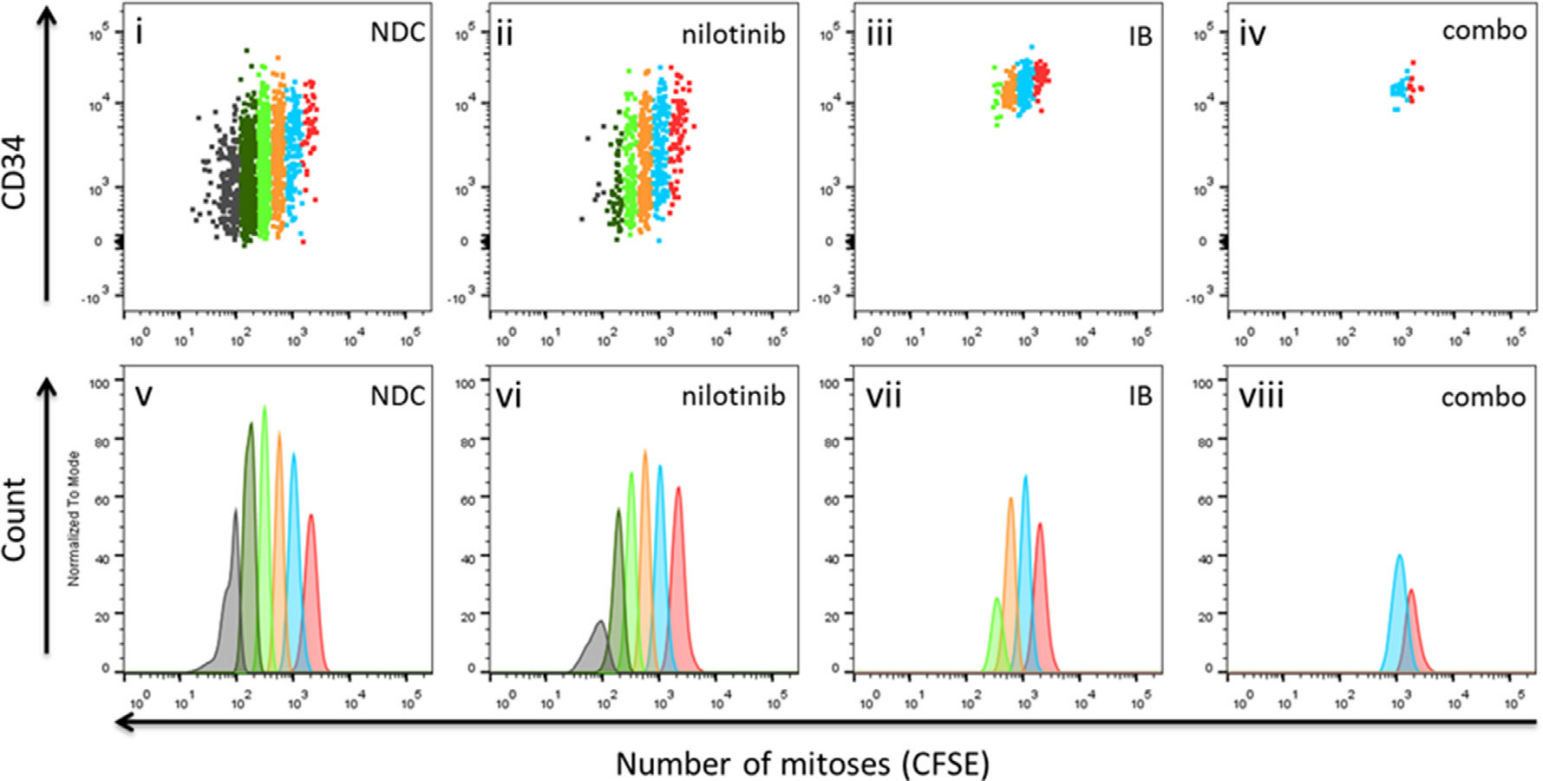
Cell division tracking of primary CML-CP cells treated *in vitro* with imipramine blue (IB) alone or in combination with nilotinib Position of viable CFSE^max^ cells (undivided; division 0) as guided by Colcemid™ treated control, is shown in red in all panels. Cells harvested from the dish having divided once are shown in blue, having undergone two divisions are in orange, in division 3 in bright green, in division 4 in dark green, and divided 5 times or more (CFSE^negative^ cells) in gray. Input was 100 × 10^3^ total CML388 CD34^+^ cells per well. Cells were cultured for 72 h in serum free medium with physiological growth factors with or without nilotinib (1 μM) and /or IB (1 μM). It can be seen that the few cells remaining alive after IB treatment, alone or in combination with nilotinib, were in early divisions with higher CD34 surface expression.

We have previously shown that TKI can hold CD34^+^ CML-CP cells out of cycle *in vitro* without killing them [21]. To determine if this ‘anti-proliferative' phenomenon was also true for IB, cell division tracking was performed using the vital fluorescent stain, CFSE then assaying surviving cells for colony forming ability (CFC). After 72 h in culture, CFSE loaded CML-CP cells were stained with anti-CD34-APC and the cells in the viable gate (as assessed by FSC/SSC) were analysed for cell division history. Firstly, it could be seen that with CML cells, the total number of live events was reduced with IB treatment with respect to either nilotinib or DMSO (NDC) control, and more so with the combination (Figure 4; *p* = 0.006 combination v NDC). It may also be inferred that the cells remaining ‘alive' after IB treatment were more primitive i.e. in an early division by CFSE with higher CD34 surface expression, than was seen with nilotinib treatment. However drug treatment had not enriched for CML stem/progenitor cells as the remaining few were non-functional primitive cells, as shown by compromised clonogenicity capacity in colony forming cell (CFC) assay (c.f. Figure 3D).

From these cell division tracking experiments, an anti-proliferative effect in CML, that is restriction of cell division by IB alone and in combination with nilotinib may be presumed from the higher percentage of cells recovered in lower divisions in either of these drug conditions compared to no drug control (NDC) or nilotinib alone (Table 2; Supplemental Table S1). However, from the absolute number of cells recovered from the wells, calculated as the product of the percentage in gate multiplied by total viable cell count, with IB alone or in combination with nilotinib, the synthetic lethality is evident, that is while either drug alone may be compatible with viability, the combination of both leads to cell death.

**Table 2:** Percentage of cells and absolute cell numbers recovered by division

CML388	Viable cells (percentage in gate and absolute cell number)							
Division #	0		1		2		3, 4, 5	
Treatment	%	cell no.	%	cell no.	%	cell no.	%	cell no.
*NDC*	2.0	41	7.5	155	16.2	337	61.5	1276
*nilotinib (1 μM)*	10.5	74	22.5	159	29.2	206	24.8	175
*IB (1 μM)*	13.5	38	49.6	140	24.8	70	4.3	12
*Combination*	28.9	11	63.2	24	0	0	0	0

From these cell division tracking experiments, an anti-proliferative effect in CML that is restriction of cell division, of IB alone and in combination with nilotinib may be presumed from the higher percentage of cells recovered in lower divisions in either of these drug conditions compared to No Drug Control (NDC) or nilotinib alone. However, from the absolute number of cells recovered from the wells, calculated as the product of the percentage in gate multiplied by total viable cell count, with IB alone or in combination with nilotinib, the synthetic lethality is evident, that is when either drug alone may be compatible with viability but the combination of both leads to cell death.

### BCR-ABL1^negative^ CD34^+^ cells retain their primitive function with IB and nilotinib combination treatment

**Figure 5:**
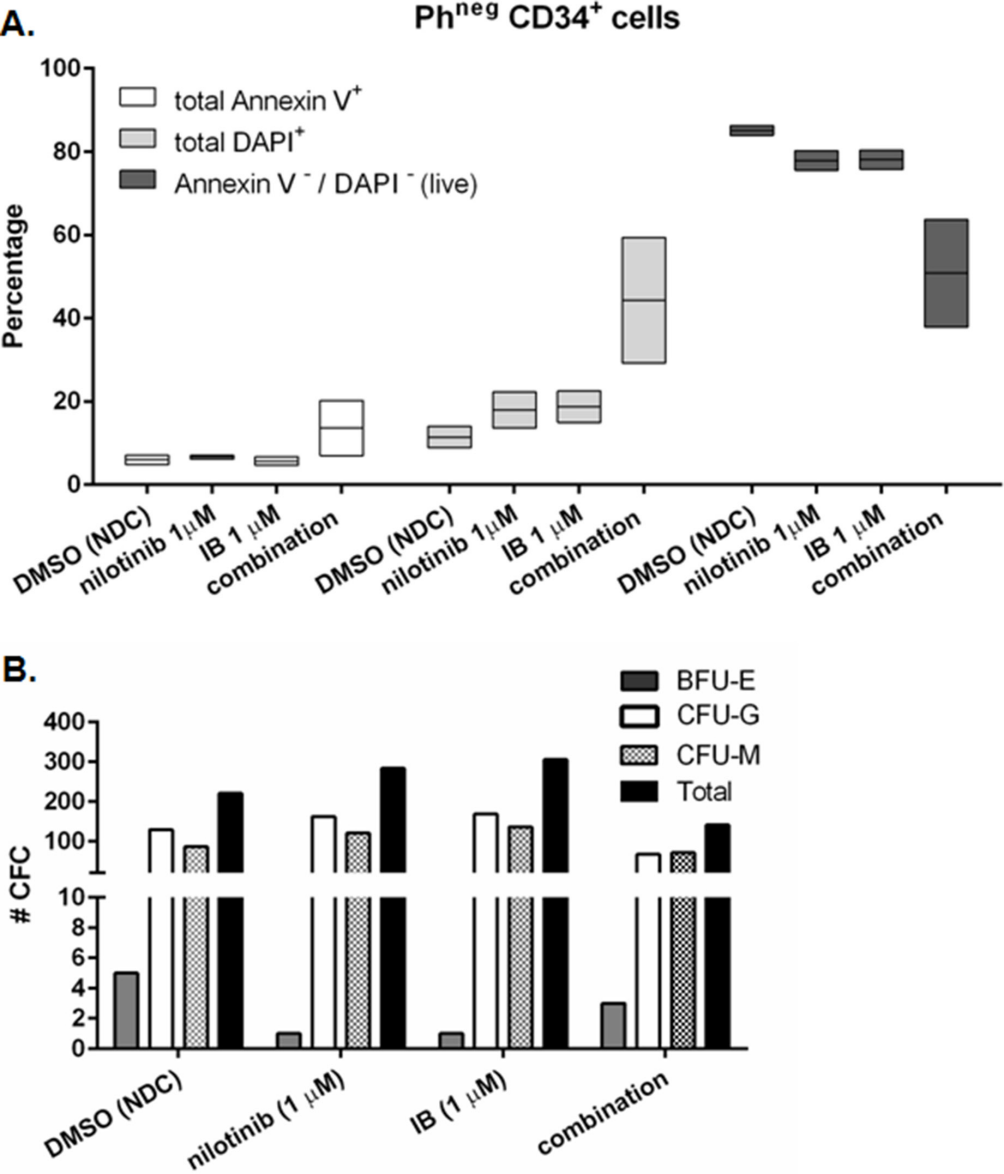
Response of BCR-ABL1^negative^ CD34^+^ cells to imipramine blue (IB) in combination with nilotinib (**A**) Primary BCR-ABL1negative (normal) CD34+ cells from two donors were treated in culture with nilotinib or IB alone, or nilotinib plus IB in combination. After 72 h, cells were stained with Annexin V and DAPI to assess apoptotic response to the drug(s) by flow cytometry. White boxes: total Annexin V^+^ (dead and dying); light grey boxes: total DAPI^+^ (dead); dark grey boxes: total Annexin V^−^ and DAPI^−^ (most viable). (**B**) After 72 h drug treatment, cells were harvested and plated into semi-solid culture medium for 12 days after which time colony forming cells (CFC) were scored for number of individual colonies and type. ‘#CFC' is the mean of two plates per condition. No significant differences in total number of CFC were seen between treatment arms, or with respect to no drug control (NDC) by Student's *t*-test. One representative result is shown.

We next needed to know if normal hematopoiesis may be affected by IB, so we modelled this *in vitro* using BCR-ABL1^negative^ CD34^+^ cells which are immature hematopoietic cells lacking the causative oncogenic driver translocation. Gating on Annexin V/DAPI double negative events for viable cells, the percentage live cells remaining in the well after treatment of BCR-ABL1^negative^ (normal) CD34^+^ cells with 1 μM each of nilotinib and IB, was approximately half the untreated control (e.g. 84 v 38%; Figure 5A) which was a greater surviving fraction than seen with treated CML-CP cells (1%, c.f. Figure 3A; *p* = 0.001 CML versus normal). Moreover, the functional capacity (ability to form a colony in CFC assay) of surviving normal CD34^+^ cells was unaffected by either drug alone or combination (Figure 5B) whereas for CML-CP cells there were16 times fewer total CFC with the combination treatment compared to normal; average 4% (CML) versus 64% (normal) of respective NDC.

### IB blocks NF-κB activation in BCR-ABL1^+^ cells

**Figure 6:**
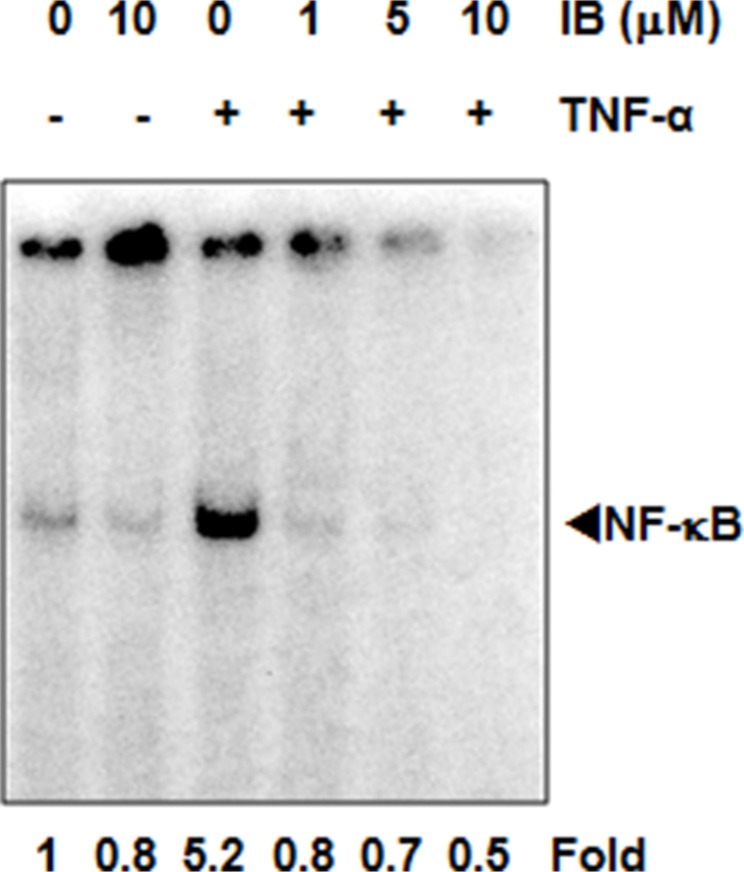
Imipramine blue (IB) blocks NF- κ**B activation in Ph^+^ cells.** KBM-5 (2 × 10^6^/mL) cells treated with IB for 12 h and then exposed with 0.1 nM TNF-α for 30 minutes. Cells were harvested, nuclear extract prepared and EMSA performed. Numbers below figure are fold changes relative to the untreated control.

To determine whether IB inhibited key intracellular signaling pathways, we first examined the effect of IB on cytokine-induced activation of NF-κB. TNF-α led to prominent induction of NF-κB as assessed by electrophoretic mobility shift assay (EMSA) in BCR-ABL1^+^ KBM-5 cells (Figure 6). Pre-treatment with IB led to a concentration-dependent decrease in activated NF-κB, with a greater than 60% inhibition at 10 μM (Figure 6). A similar concentration-dependent decrease in MAP kinase activation was also seen.

### IB significantly increases PP2A activity in 32D-BCR-ABL1 cells

Reactive oxygen species are known to inactivate many protein phosphatases through oxidation of sulfhydryl groups [18]. Because IB inhibits NADPH oxidase, we sought to investigate whether IB-induced apoptosis of CML stem/progenitor cells depends, at least in part, on inhibition of PP2A tumor suppressor activity as we previously reported in both TKI-resistant quiescent stem and progenitor cells from CML-CP and -BC patients [7, 19].

**Figure 7:**
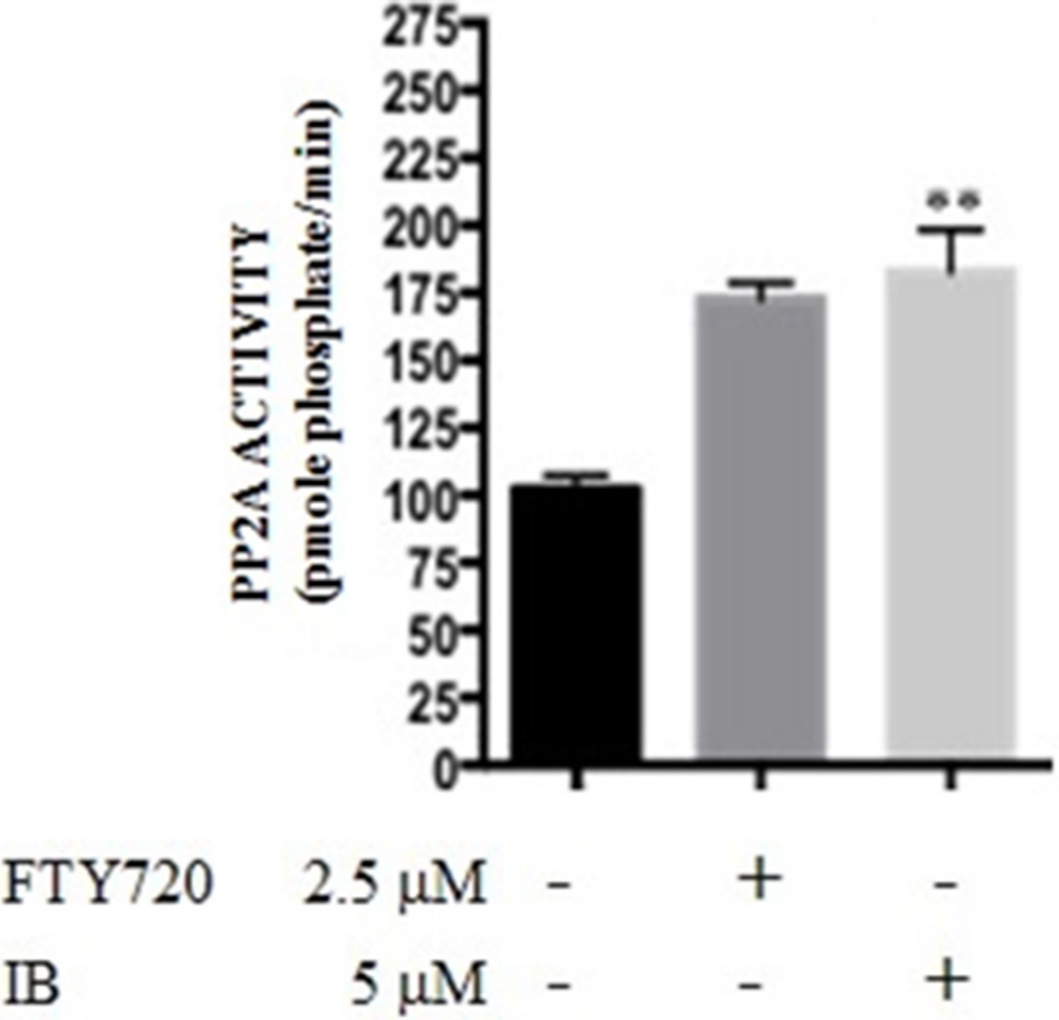
Imipramine blue (IB) acts as a PAD in 32D-BCR-ABL cells PP2A phosphatase activity (pmole phosphate/min) was assessed in untreated 32D-BCR-ABL cells and upon 6 h exposure to IB at concentration of 5 μM. The PAD, FTY720 (2.5 μM), was included as a positive control. Student's *t* tests were performed using GraphPad Prism version 6.0a with a *P* value < 0.05 being considered significant.

To determine whether IB acts as a PP2A-activating drug (PAD) [8], PP2A phosphatase activity was assessed in 32D-BCR-ABL1 myeloid cells exposed for 6h to IB (5 μM) (Figure 7). Untreated 32D-BCR-ABL1 cells were used as negative controls [19]. Treatment with IB restored PP2A activity in 32D-BCR-ABL1 cells (Figure 7) at levels higher than those observed in parental 32Dcl3 cells (not shown) and upon treatment of 32D-BCR-ABL1 cells with the potent PAD, FTY720 [9] (Figure 7). Thus, IB seems to exert its anti-leukemic activity, at least in part, through reactivation of PP2A.

**Figure 8:**
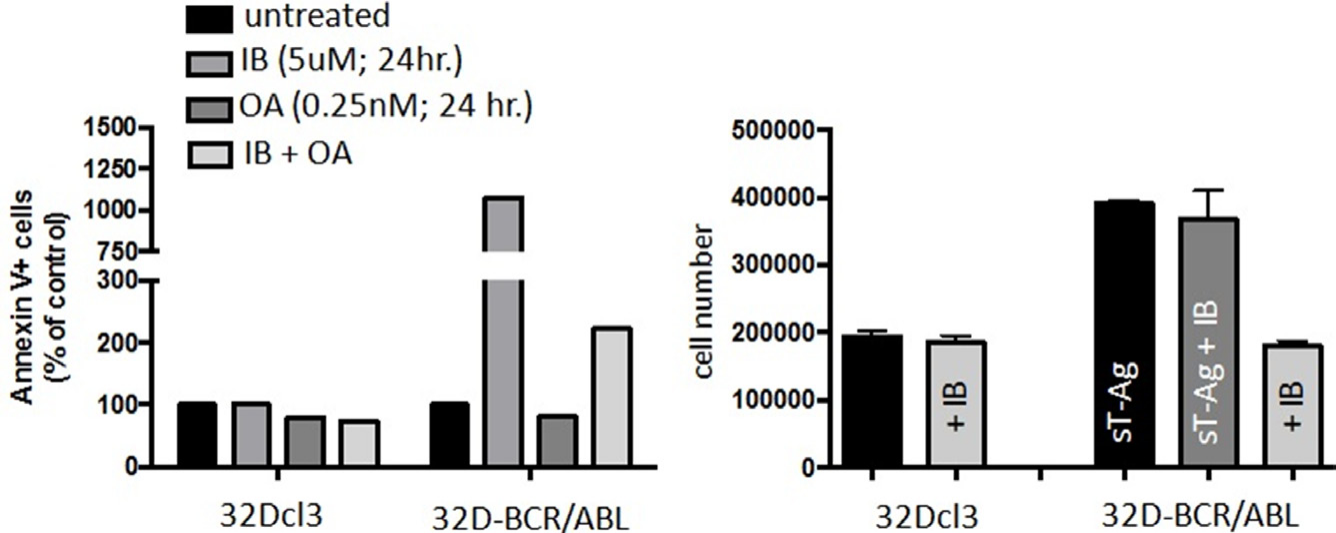
PP2A mediates the pro-apoptotic activity of imipramine blue (IB) in 32Dcl3 and 32D-BCR-ABL1 cells (**A**) Effect of IB on viability of wild type and small T antigen (sT-Ag)-expressing 32D-BCR-ABL1 cells. Note that both 0.25 nM OA treatment and sT-Ag expression results in suppression of PP2A phosphatase activity. (**B**) IB pro-apoptotic activity, assessed as percentage of AnnexinV^+^ cells, in parental and BCR-ABL1-expressing 32Dcl3 exposed to 0.25 nM of OA, and combination. Student's *t* tests were performed using GraphPad Prism version 6.0a with a *P* value < 0.05 being considered significant.

In order to mechanistically show a role of PP2A, we treated cells with okadaic acid (OA), used at a concentration (0.25 nM) that reportedly inhibits PP2A activity only [9, 19], as well as genetically inhibiting PP2A by expressing the small T antigen (sT-Ag). Treatment with the PP2A inhibitor OA blunted the response to IB, while sT-Ag nearly completely blocked the response to IB. Thus, PP2A reactivation likely plays a major role in the activity of IB (Figure 8A).

To determine the pro-apoptotic activity of IB in both parental and BCR-ABL1-expressing 32Dcl3 cells, cells were incubated with the drug *in vitro* and viable cell counts performed after 24h using Annexin V staining (Figure 8B). The cells were treated with 5 μM of IB, 0.25 nM of OA, and combination.

## DISCUSSION

CML was the first malignancy to be associated with a specific translocation, namely the Philadelphia (Ph) chromosome associated t(9;22) translocation. The ability of the BCR-ABL1 to serve as an oncogenic driver mutation has been elegantly described in both transplantation experiments and transgenic models. The causality of BCR-ABL1 in myeloid malignancy served as the impetus to develop specific inhibitors of BCR-ABL1, beginning with imatinib, and followed with additional inhibitors to target both wild type and a multitude of mutant BCR-ABL1, including BCR-ABL1^T315I^. The discovery of leukemia stem cells that are resistant/insensitive to TKI has informed us of the need to target this population to obtain cure [20–22]. Nieborowska-Skorska *et al*., have described elevated levels of superoxide in the CD34^+^CD38^−^ CML precursor cells [11], thus establishing reactive oxygen signaling as a legitimate target in eliminating CML stem cells [7]. Indeed poor responders to TKI therapy accumulated more ROS, 8-oxoG, and γ-H2AX than good responders. Accumulation of ROS was independent of proliferation, as elevated levels of ROS were found in dormant leukemia cells [11]. Further, elevated levels of ROS are not fully normalized by TKI and have been shown to cause hypermethylation of tumor suppressor genes such as p16ink4a [2, 16].

Treatment with targeted therapies has been widely heralded in the clinic, but has also informed us that advanced malignancy is not “addicted” to a single oncogene or signaling pathway. Both intrinsic and extrinsic modes of resistance have been described, and redundant as well as multiple signaling pathways observed in resistant cells. The continued use of targeted therapies likely selects for the expansion of a stem cell population resistant to therapies and capable of causing rapid recurrence, either in the presence of targeted therapy, or once it is discontinued. The relapse rate of patients treated with long term TKI has been evaluated in the STIM and TWISTER studies. In the TWISTER study, patients with undetectable minimal residual disease were allowed to discontinue TKI, and a minority (47%) did not relapse at 24 months [23]. Similarly in the STIM study, discontinuation of TKI led to the majority of patients relapsing before 6 months [24]. These studies are in the 10–20% of patients with the most favorable response; that is CMR for CML. For patients with residual disease, discontinuation of TKI is not advised, and these patients may have acquired additional mutational burden preventing a CMR. Elimination of this population is thus an unmet need in treatment of hematopoietic malignancy, and the only treatment that has eliminated the stem cell population is bone marrow transplantation. Even allogeneic bone marrow transplantation, a highly morbid procedure, does not guarantee freedom from relapse.

**Figure 9:**
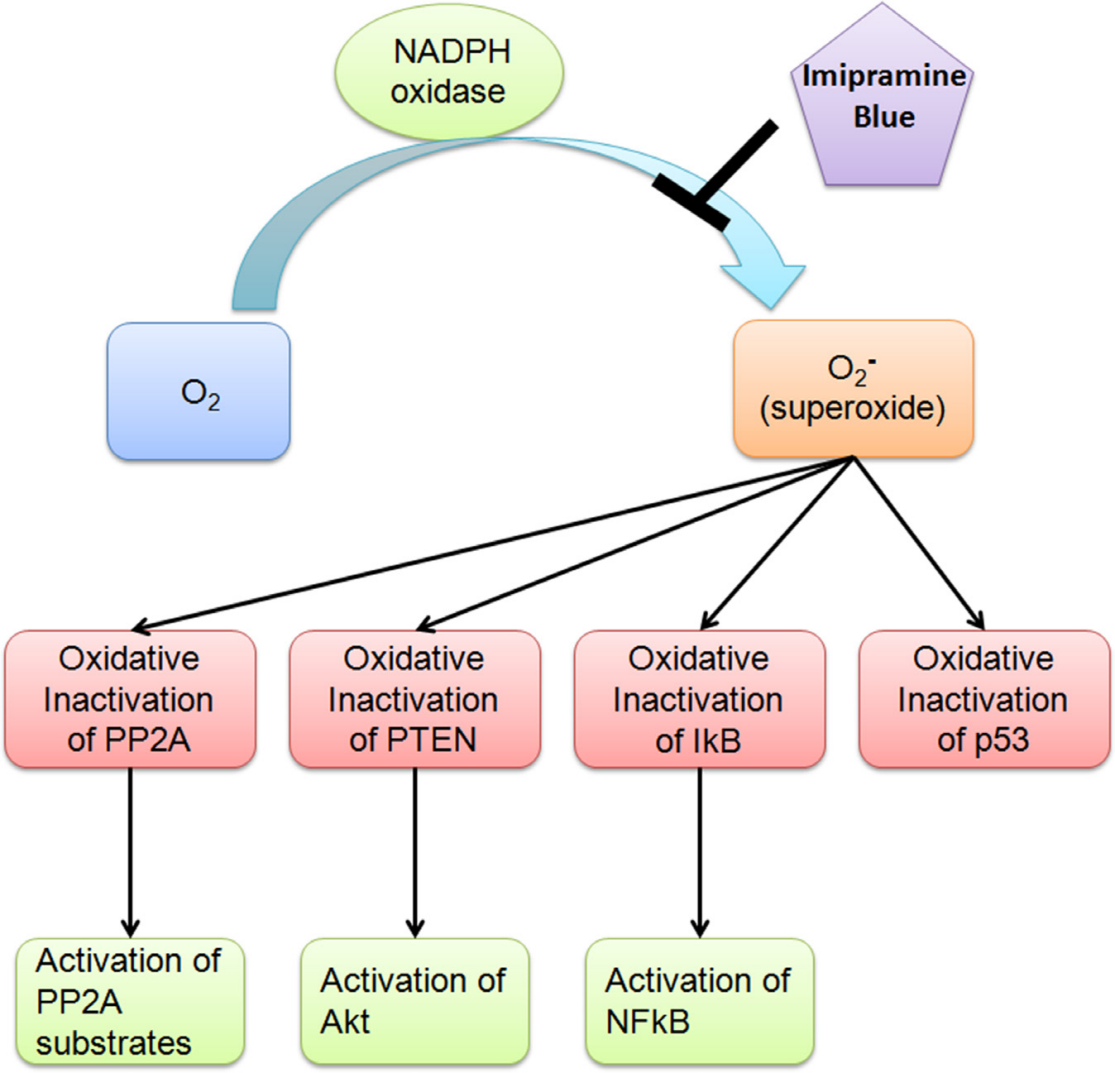
Proposed mechanism of NADPH oxidase Elevated levels of ROS in neoplastic stem cells, which is not normalised by TKI and in contrast to low levels of ROS in normal HSC, may provide a therapeutic window.

Recent data points towards the signaling pathways necessary for the maintenance of leukemic stem cells. While cancer stem cells have been shown to have either low or elevated levels of ROS, the role of ROS in stem cell maintenance appears to be context dependent. In the case of myeloid leukemia, the balance of the data supports elevated levels of ROS favoring a stem cell phenotype and resistance to targeted therapies. Of interest, normal HSC in an osteoblastic niche appear to have a phenotype of low levels of ROS, elevated levels of notch 1 and N-cadherin, with low levels of p38 MAP kinase, p16ink4a and p53 [3]. In a model of bone marrow failure in ataxia telangiectasia (Atm) null mice, bone marrow exhaustion was associated with upregulation of p38MAPK as a reactive oxygen response [25]. BCR-ABL1 itself causes increased generation of ROS that causes upregulation of Fyn kinase, which may also mediate resistance to targeted therapies [13]. Thus, in Ph^+^ myeloid malignancy, the elevated levels of ROS in neoplastic stem cells, which is not normalised by TKI and in contrast to low levels of ROS in normal HSC, may provide a therapeutic window (Figure 9) [2, 14, 17]. This difference between normal and malignant stem cells may be targeted with an NADPH oxidase inhibitor. Prior gene analyses of CML stem cells versus normal HSC revealed differences in cell cycle genes despite similar levels of quiescence [26]. This may be a function of differing baseline levels of ROS, with normal HSC being driven towards maturation and senescence on elevation of ROS, while elevated levels of ROS may favor survival and NF-κB elevation in tumor stem cells.

Mechanistically, we found that IB acts as a potent PAD [8] as it was capable of strongly reactivating the enzymatic activity of the PP2A tumor suppressor that, reportedly, is inactive in both CML quiescent stem and proliferating progenitor cells [7, 19], and so inhibiting NF-κB [15, 27]. Indeed, the pro-apoptotic activity of IB and its ability to potentiate the effect of TKIs, is consistent with the effect of other PADs in TKI-resistant CML stem and progenitor cells [7, 8, 28]. The role of PP2A in mediating the effects of IB was further elucidated both pharmacologically by using OA at a concentration that inhibits PP2A activity only [29], and genetically, with sT-Ag, a well studied inhibitor of PP2A that binds PP2A catalytic subunit and prevents its assembly into a functional multimeric phosphatase [30]. Treatment of 32D-BCR-ABL cells with OA blunted the apoptotic response to IB, and introduction of sT-Ag nearly completely blocked the apoptotic response to IB. This suggests that PP2A reactivation plays a major role in the activity of IB.

The therapeutic role of NADPH oxidase inhibitors in hematopoietic disorders is not fully understood. In our previous study of the NADPH oxidase inhibitor IB in solid tumors, we saw striking inhibition of invasion of GBM into the brain [31]. As a single agent, IB did not significantly extend the lifespan of rats but when combined with the conventional chemotherapeutic agent doxorubicin, long term survival was noted in all treated animals. A potential mechanism of such long term survival is the targeting of tumor stem cells, which are resistant to conventional chemotherapy alone. This finding illustrates a potential cardinal principle in the use of NADPH oxidase inhibitors; whilst they may not be highly effective as monotherapy, in combination with a targeted agent and/or conventional chemotherapy, their potency is realized. In leukemia, this may in part reflect the complex microenvironment in which macrophages, locally produced growth factors, and stromal cells support neoplastic leukemia cells [32–40].

Signaling in tumor stem cells is associated with elevated levels of NF-κB signaling [41, 42], which prevents tumor apoptosis at a number of levels, including increased expression of drug efflux pumps, as well as production of inflammatory cytokines that promote stromal-tumor interactions. Increasing evidence from the clinic demonstrates that tumors with stem cell characteristics, such as CML, gain independence from original driver mutations. So once again CML is a paradigm for cancer therapy highlighting the need for complementary agents targeting alternative signaling pathways. We demonstrate that IB, an agent in preclinical development, kills CML cells with stem cell characteristics. Combination therapy of TKI and IB may be of benefit in the treatment of CML as well as other ROS-driven hematopoietic malignancies with driver oncogenic fusions and associated genomic instability, through targeting NADPH oxidase.

## MATERIALS AND METHODS

### Primary human cell samples

Primary cells were obtained with informed consent according to the Declaration of Helsinki, from peripheral blood or leukapheresis product of patients newly diagnosed with CML in CP, or lymphoma patients without bone marrow involvement as BCR-ABL1^negative^ normal controls. Cells were either first enriched by positive selection by magnetically activated cell separation (CliniMACS) to > 90% CD34^+^ cells or immediately cryopreserved as total mononuclear cells. Once the latter were thawed, viable cells were enriched to > 90% CD34+ cells after staining with anti-CD34 antibody by fluorescence activated cell sorting using a FACSAria flow cytometer (BD Biosciences). The study had approval from the West of Scotland Research Ethics Committee 4.

### Cell lines

KCL22, K562, KBM-5, HL60 cell lines are widely published *in vitro* models of human leukemia, their selection for use being dependent on availability in a particular laboratory. In this study, KCL22WT, K562 and HL60 were commercially sourced from cell culture collections (ECACC or DMSZ German Collection of Microorganisms and Cell Cultures). KCL22^T315I^ cells were kindly provided by Dr Nicholas J. Donato (University of Michigan) [43]. The parental human BCR-ABL1^+^ CML-BC cell line, KCL22 [44], derived from the pleural effusion of a 32 year old female patient, is hyperdiploid karyotype with 3.3% polyploidy (DSMZ #ACC 519). Basophilic, erythroblastic hypotriploidy K562 cell line was derived from the pleural effusion of 53 year old female patient in BC with genomic mutations in p53 and CDKN2a according to the Sanger COSMIC database (DSMZ #ACC 10) [45]. KBM-5 cells derived at the MD Anderson from a 67 year old female patient carries multiple copies of the Ph and chromosomes 7, 8, 15, and 17 [46]. HL60 (#ACC 3) is of hypotetraploid karyotype with 1.5% polyploidy derived from the peripheral blood of a 36-year-old female with acute promyelocytic leukemia [47].

### Cell culture

Primary human cells were cultured *in vitro* in physiological (for CML) or high (for normal) concentration growth factor (GF)-supplemented serum-free medium as previously described [26]. Cell lines (KCL22^WT/T315I^, K562, KBM-5) were cultured in RPMI1640 supplemented with 10% fetal bovine serum (FBS), 50 U/mL penicillin, 50 mg/mL streptomycin, and 2 mM L-glutamine (Invitrogen) at 37°C, 5% CO_2_. The 32D-BCR-ABL, 32D-BCR-ABL1-GFP–sT-Ag and parental 32Dcl3 mouse myeloid progenitors were maintained in culture in Iscove's modified Dulbecco medium (IMDM), 10% FBS, and 2 mM L-glutamine, 2 ng/mL murine IL-3, 50 U/mL penicillin and 50 mg/mL streptomycin.

### Flow cytometry (apoptosis, cell division tracking)

Following treatment with various concentrations of IB and/or nilotinib *in vitro*, primary CML-CP cells were harvested, surface stained with anti-CD34-allophycocyanin (APC) for maturity followed by Annexin V-phycoerythrin (PE) and propidium iodide or 4′,6-diamidino-2-phenylindole (DAPI) as markers of apoptosis, and then acquired using a FACSCanto II flow cytometer (BD Biosciences). For cell division tracking, cells were first stained with carboxyfluorescein isothiocyanate (CFSE) as previously described [48].

After 24 h drug treatments, parental/BCR-ABL1 expressing 32Dcl3 cells were washed once in PBS, then once in Annexin V binding buffer before resuspending to 1 × 10^6^ cells/mL. Five microlitres of fluorochrome-conjugated Annexin V was added per 100 μL of cell suspension followed by 15 minutes incubation at room temperature then reading by BD LSR II cell analyzer.

### Colony forming cell assay

Clonogenicity of surviving cells was assayed in semi-solid culture medium (MethoCult H4034 Optimum, Stem Cell Technologies) for 12 days. Briefly, the methylcellulose was seeded with a fixed volume or 3,000 viable cells and plated onto 35 mm culture dishes. Colonies were counted and scored as colony forming unit (CFU)-erythroid (E), CFU-granulocyte, -macrophage, -granulocyte/macrophage (G, M, G/M), CFU-granulocyte, erythrocyte, macrophage, megakaryocyte (GEMM) or burst forming unit-erythroid (BFU-E).

### PP2A assay

PP2Ac assays from whole cell lysates were carried out as described [9] using the PP2Ac immunoprecipitation (IP) phosphatase assay kit (Millipore). Briefly, protein lysate (50 μg) in 100 μL of 20 mM Hepes, pH 7.0/100 mM NaCl, 5 μg of PP2Ac antibody (Millipore), and 25 μL of Protein A–agarose were added to 400 μL of 50 mM Tris, pH 7.0/100 mM CaCl_2_, and IPs were carried out at 4°C for 2 h. IPs were washed and used in the phosphatase reaction according to the manufacturer's protocol. As an internal control, the amount of IP PP2A was also monitored by anti-PP2Ac Western blots (data not shown). Both OA treatment and sT-Ag expression as procedures to inhibit PP2A activity have been previously described [9, 19].

### Electrophoretic mobility shift assay (EMSA)

To assess NF-κB activation, we isolated nuclei from KBM-5 cell lines and carried out EMSA essentially as previously described [22]. In brief, nuclear extracts prepared from KBM-5 cells (2 × 106/mL) were incubated with ^32^P-end-labeled 45-mer double-stranded NF-κB oligonucleotide (4 μg of protein with 16 fmol of DNA) from the HIV long terminal repeat (5′-TTGTTACAAGGGACTTTCCGCTG GGGACTTTC CAGGGA GGCGT GG-3′) for 30 min at 37°C. The resulting DNA–protein complex was separated from free oligonucleotides on 6.6% native polyacrylamide gels. The dried gel was exposed to x-ray film and quantitated by Image J software.

## SUPPLEMENTARY MATERIALS FIGURES AND TABLES


