# Supplementary material for: Cooperation of imipramine blue and tyrosine kinase blockade demonstrates activity against chronic myeloid leukemia
Source: Oncotarget. 2016 Jul 12;7(32):51651–64. doi: 10.18632/oncotarget.10541 (PMC5239504; doi:10.18632/oncotarget.10541)
Supplement: Supplementary file 1 [file oncotarget-07-51651-s001.pdf]

Cooperation of imipramine blue and tyrosine kinase blockade demonstrates activity against chronic myeloid leukemia

Supplementary Materials

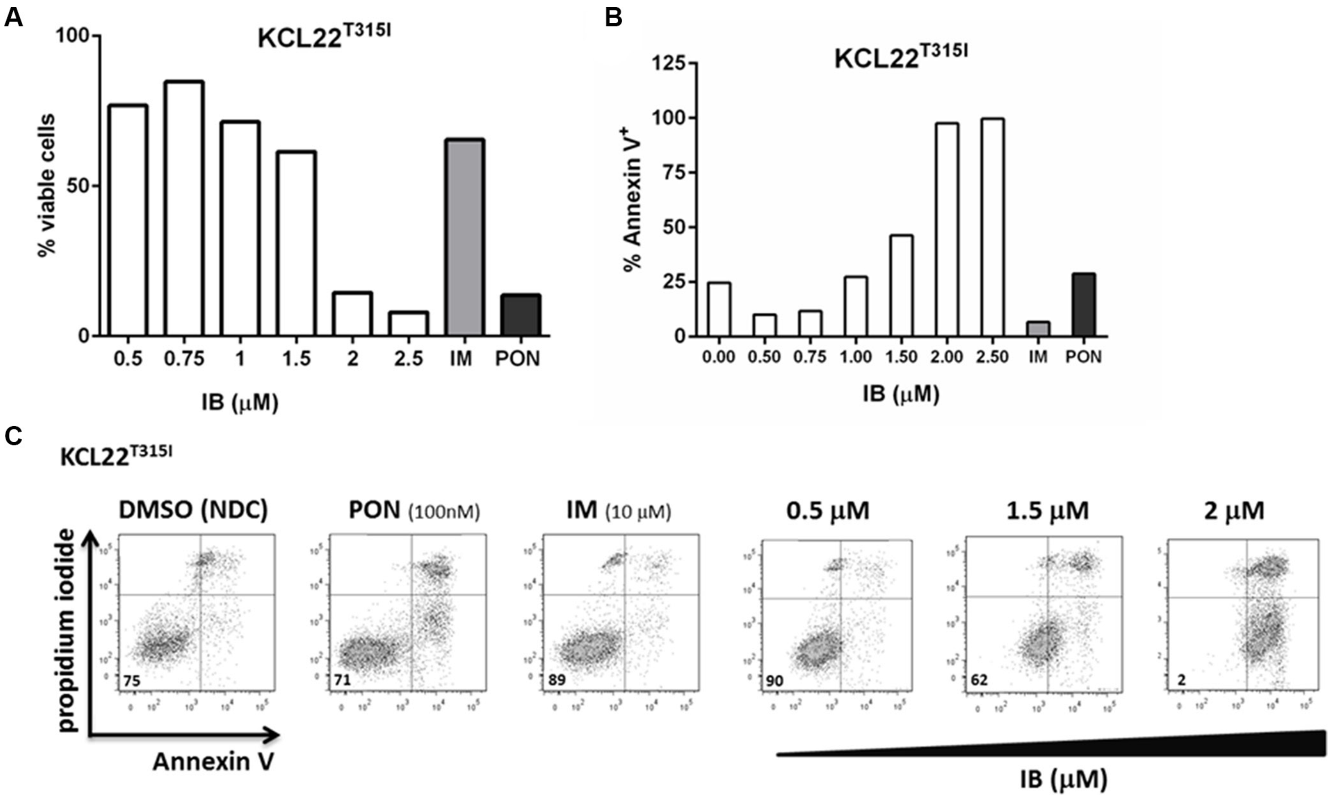

Supplementary Figure S1: (**A**) The number of viable BCR-ABL1-mutated KCL22^{T315I} cells remaining after 72 h treatment with IB were counted by Trypan Blue dye exclusion method and normalized to the untreated control (100%) and (**B**) stained with Annexin V and propidium iodide to assess apoptotic response to the drug by flow cytometry. Imatinib (IM, 10 μM) and ponatinib (PON, 100 nM) were used as 'negative' and 'positive' controls, respectively (A and B; *n* = 1, duplicate wells). **(C)** Illustrative flow cytometry dot plots of KCL22^{T315I} cell response to IB, IM, or PON after 72 h.

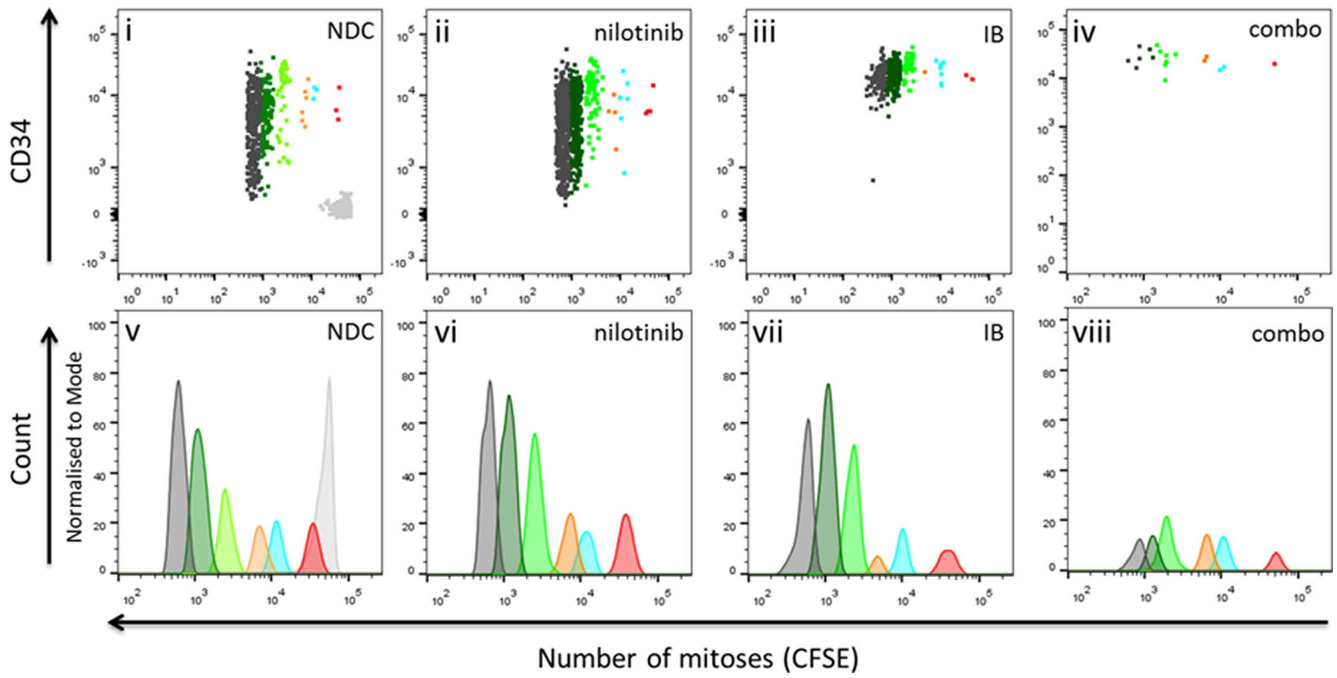

Supplementary Figure S2: Cell division tracking of CML-CP cells treated *in vitro* with imipramine blue (IB) alone or in combination with nilotinib. Position of CFSE^{max} cells on day zero is shown in light grey in panels (i) and (v). Position of undivided (division 0) cells after 72 h in culture is shown in red in all panels. Input was 80 × 10^3 total CML391 CD34$^+$ cells per well. Cells were cultured in serum free medium with physiological growth factors with or without nilotinib (1 µM) and /or IB (1 µM).

Supplementary Table S1: Percentage of cells and absolute cell numbers recovered by division

**CML391**	**Viable cells (percentage in gate and absolute number)**							
Division #	***0, 1, 2***		***3***		***4***		***5***	
Treatment	***%***	***cell no.***	***%***	***cell no.***	***%***	***cell no.***	***%***	***cell no.***
NDC	0.7	12	2.1	37	6.8	123	18.4	331
nilotinib (1 µM)	0.8	14	4.9	84	15.5	267	25.1	433
IB (1 µM)	2.4	10	9.8	41	44.9	188	33.2	139
Combination	26.3	5	36.8	7	10.5	2	21.1	4

From these cell division tracking experiments, an anti-proliferative effect in CML, that is restriction of cell division, of IB alone and in combination with nilotinib may be presumed from the higher percentage of cells recovered in lower divisions (from undivided (division 0, $CFSE^{max}$) to division 2) in either of these drug conditions compared to No Drug Control (NDC) or nilotinib alone. However from the absolute number of cells recovered from the wells with IB alone or in combination with nilotinib, the synthetic lethality is evident. Input numbers: 8×10^4 CML391 $CD34^+$ cells per well. Total number of viable cells recovered from NDC was 1803 cells, 1725 cells from nilotinib treated well, 419 cells from IB well, and with combination, only 19 cells.
